# A Workshop on Writing Letters to the Editor

**DOI:** 10.15694/mep.2020.000006.1

**Published:** 2020-01-07

**Authors:** Yuki Kataoka, Azusa Sakurai, Hideki Mori, Hideto Yoshida, Yasushi Nakano, Kotaro Fujii, Rie Matsushita, Ryuji Suzuki, Ryuto Shiraishi, Toshihiko Takada

**Affiliations:** 1 Hyogo Prefectural Amagasaki General Medical Center; 2 National Hospital Nagasaki Medical Center; 3 Nishiizu Ken-iku-kai Hospital; 4 Kawasaki Municipal Ida Hospital; 5 Shirakawa Satellite for Teaching And Research (STAR); 6 Kikugawa General Hospital

**Keywords:** scholarly activity, academic writing, workshop, Japan

## Abstract

This article was migrated. The article was marked as recommended.

**Background:** Writing letters to the editor based on critical appraisal can serve as the first step in scholarly activity. The workshop in this study focused on educating physicians about the best ways to write letters to the editor.

**Methods:** We conducted a 90-minute workshop as a part of scientific conference. Participants were physicians and medical students who chose to join this workshop. We developed the following learning outcomes for participants: 1) to be able to explain falsificationism; 2) to be able to explain how to check author instructions; 3) to be able to explain how to write a letter to the editor.

**Results:** Twenty-eight participants, including three medical students, attended the workshop. Participants’ satisfaction with the workshop had a mean of 4.5 points out of 5 (standard deviation: 0.5). Nearly 80% of participants achieved the learning outcomes. Four participants’ groups submitted letters within a month after the workshop, and all four were published. These four groups encompassed a total of 13 authors. In addition, none of the first author of each letter had previously written a clinical research paper.

**Findings and Discussion:** This workshop improved not only the participants’ knowledge but it also led to the concrete result of four published letters. Japanese physicians would be able to use this framework to write letters to the editor.

## Introduction

Japanese physicians face difficulties in conducting clinical research. One of the reasons is the lack of knowledge in designing and conducting clinical research. In undergraduate and postgraduate education, or continuing professional development, most Japanese physicians are untrained for clinical research. Some exceptions are the basic laboratory training received by undergraduates, or when students opt to study for a PhD after graduation ( Fukuhara S, Sakushima K, 2012). In the past, many physicians went to graduate school to learn research methods, but in recent years, fewer doctors have followed this path. One of the reasons is that graduate students are forced to work for very low pay, or for no pay at all, at university hospitals ( Shibuya and Unno, 2019).

One solution would be to equip a large number of non-academic physicians without research skills, as they are interested in the research itself ( Kurita *et al.*, 2016). We have conducted various workshops with the aim of increasing research output. In this article, we would like to introduce the contents of a workshop for beginners and share the lessons learned from it.

Writing letters to the editor based on critical appraisal can serve as the first step in scholarly activity. The reasons are as follows. First, critical appraisal of existing research is essential when developing a new clinical study. Second, novices can easily gain experience of journal submission. Third, the time it takes to write a letter, and its submission process, is shorter than it is for original research. However, there are few opportunities to learn how to write these letters in Japan ( Kataoka *et al.*, 2018). Therefore, the aim of the workshop was to educate physicians about how to write letters to the editor.

## Methods

We conducted this workshop as a part of the Annual Meeting of the Japan Chapter of the American College of Physicians in Kyoto, Japan, in 2018. Participants were physicians and medical students who chose to join this workshop. Workshop facilitators included four residents, four prior-participants of this workshop, and two Master of Public Health. They were all Japanese physicians.

### Learning Outcomes

We developed the following learning outcomes:

• To be able to explain falsificationism
• To be able to explain how to check the author instructions
• To be able to explain how to write a letter to the editor

### Workshop program development

We shortened a two-day program for physicians based on an existing two-year distance-learning program that was designed to teach the skills necessary to conduct clinical studies ( Kataoka *et al.*, 2018). To shorten the two-day workshop to 90 minutes we used a flipped classroom framework ( Chen *et al.*, 2018). In the video shown to participants before the workshop, we explained falsificationism and how to write a letter to the editor using a risk of bias tool. Falsificationism is a scientific philosophy proposed by Karl Popper. The core idea of falsificationism is that a hypothesis must be falsifiable to be scientific ( Popper, 2005). We adopted this concept for the workshop as follows. Published articles contain hypothesis. Articles are refined by letters from readers in addition to peer reviews before publication.

We selected and shared with participants an article published in *Annals of Internal Medicine* the month before the workshop took place ( Graham *et al.*, 2018). The reason why we selected the article was published online ahead of print which extended the deadline to submit letters. Participants evaluated this article before the workshop.

One instructor conducted a pilot test with the same content. Three attending physicians participated. For the sake of clarity, we modified the actual lecture slides slightly based on the personal communication with participants.

We show the final program in Table 1.

**Table 1.** Structure of the workshop for physicians on writing letters to the editor

Timing	Time	Content
Prior to workshop	13 min	Pre-class movie Falsificationism Checking “author information” Example of a “letter to the editor” https://youtu.be/mQ8BXtw_cVI
	About 60 min	Read the article *
Workshop	15 min 20 min 10 min 20 min 15 min 5 min	Lecture: Risk of bias and confounding Groupwork: Sharing critiques Lecture: Example of a “letter to the editor” How to write in English Groupwork: Writing a letter in Japanese Presentation of each group’s work Wrap-up
Post workshop		Submission

* Graham, K. L., Auerbach, A. D., Schnipper, J. L., Flanders, S. A., *et al.* (2018), Preventability of Early Versus Late Hospital Readmissions in a National Cohort of General Medicine Patients, *Annals of Internal Medicine*,168(11), p. 766. https://doi.org/10.7326/M17-1724.

In the workshop we intended to discuss confounding and information bias ( Grimes and Schulz, 2002). We instructed the participants on how to write a letter pointing out a bias that weakened the conclusion and was not discussed in the text.

### Evaluation

We evaluated this workshop using an anonymous post-workshop questionnaire given to participants, and by the number of letters published. We followed up with the participants via email.

### Ethical consideration

The workshop received ethics approval from the Hyogo Prefectural Amagasaki General Medical Center. We received individual consent from participants to summarize the results.

## Results

Twenty-eight participants, including 3 medical students, attended the workshop and were randomly divided into 7 groups for group activities. Twenty-five participants completed the post-workshop questionnaire, while 3 attendees completed it but refused to be included in the analysis. Participants’ satisfaction with the workshop had a mean of 4.5 points (standard deviation: 0.5) out of 5. In the self-evaluation, 17 participants (77%) responded that they could now explain falsificationism; 18 participants (81%) stated that they could explain how to check author instructions; and 19 participants (86%) answered that they now could explain how to write a letter to the editor. Four participants’ groups submitted within a month after the workshop. A total of 13 authors across 4 groups ultimately had letters publishedin the *Annals of Internal Medicine* ( Table 2). In addition, none of the first authors of each letter had previously written a clinical research paper.

**Table 2.** Published letters related to the workshop

Inagaki, Y., Maruta, M., Nakano, Y. and Higuchi, J. (2019) ‘Preventability of Early Versus Late Hospital Readmissions.’, *Annals of internal medicine*, 170(3), pp. 217-218. https://doi.org/10.7326/L18-0610 Mine, A., Ikeda, S., Makiishi, T. and Matsushita, R. (2019) ‘Preventability of Early Versus Late Hospital Readmissions.’, *Annals of internal medicine*, 170(3), p. 218. https://doi.org/10.7326/L18-0608 Nagasaki, K., Akiyama, Y., Hayashi, M. and Mori, H. (2019) ‘Preventability of Early Versus Late Hospital Readmissions.’, *Annals of internal medicine*, 170(3), p. 219. https://doi.org/10.7326/L18-0609 Takata, T. and Katoka, Y. (2019) ‘Preventability of Early Versus Late Hospital Readmissions.’, *Annals of internal medicine*, 170(3), pp. 218-219. https://doi.org/10.7326/L18-0606

## Discussion

We conducted a brief workshop that was focused on educating physicians on how to write letters to the editor. Participants reported being well satisfied with the workshop, and following the workshop, approximately half of the participants had their letters published.

The strength of this workshop was that it improved not only the participants’ knowledge but it also led to the concrete result of four published letters in the *Annals of Internal Medicine* ( Kirkpatrick and Kirkpatrick, 2016). Similar short-term research workshops usually improve knowledge levels, but not results. We think that this difference was made by focusing on the letter to the editor, a type of scholarly activity that produces short-term results.

There are two areas of improvement that should be made to the design of the workshop. First, the topic of the analyzed article was difficult for Japanese participants to understand due to differences between the medical systems. Therefore, we should choose a more familiar topic for participants in future workshops. Second, we could not share information about errors in the journal submission system prior to submission, which led to submissions taking extra time. We could not share the error information immediately. The delay of information sharing for the remote follow remains a problem to be solved in future. Despite these limitations, this workshop appears to be beneficial for Japanese physicians with insufficient knowledge regarding clinical research ( Kataoka *et al.*, 2019).

Based on these initial findings, we launched an e-learning site to scale-up the project. Participants can subscribe to watch lecture videos including other scholarly activities (e.g. time management, how to write and conduct systematic review, and how to write a protocol of prediction model study). The number of participants who can receive feedback is limited due to a lack of manpower. We are recruiting participants who want to become “feedback providers” (i.e., research mentors) through the site.

## Take Home Messages

Our workshop was effective in educating Japanese physicians about how to use this framework to write letters to the editor. All of the learning outcomes established for the workshop were successful, and 13 participants across 4 groups had their letters published in the *Annals of Internal Medicine.* Further investigation to scale up this workshop is warranted.

## Notes On Contributors

Yuki Kataoka, MD, MPH, DrPH, is an attending physician at Hyogo Prefectural Amagasaki General Medical Center, Amagasaki, Japan. ORCID ID: https://orcid.org/0000-0001-7982-5213

Azusa Sakurai, MD, is a resident at Hyogo Prefectural Amagasaki General Medical Center, Amagasaki, Japan.

Hideki Mori, MD, is an attending physician at National Hospital Nagasaki Medical Center, Omura, Japan.

Hideto Yoshida, MD, is an attending physician at Nishiizu Ken-iku-kai Hospital, Nishiizu, Japan.

Yasushi Nakano, MD, PhD, is an attending physician at Kawasaki Municipal Ida Hospital, Kawasai, Japan.

Kotaro Fujii, MD, is a resident at Shirakawa Satellite for Teaching And Research (STAR), Fukushima Medical University, Shirakawa, Japan.

Rie Matsushita, MD, is an attending physician at Kikugawa General Hospital, Kikugawa, Japan.

Ryuji Suzuki, MD, is a resident at Shirakawa Satellite for Teaching And Research (STAR), Fukushima Medical University, Shirakawa, Japan.

Ryuto Shiraishi, MD, is a resident at Shirakawa Satellite for Teaching And Research (STAR), Fukushima Medical University, Shirakawa, Japan.

Toshihiko Takada, MD, MPH, PhD, is an attending physician at Shirakawa Satellite for Teaching And Research (STAR), Fukushima Medical University, Shirakawa, Japan. ORCID ID: https://orcid.org/0000-0002-8032-6224
