# Supplementary material for: A Workshop on Writing Letters to the Editor
Source: MedEdPublish (2016). 2020 Jan 7;9:6. [Version 1] doi: 10.15694/mep.2020.000006.1 (PMC10698729; doi:10.15694/mep.2020.000006.1)
Supplement: Peer review history

# Reviewer report: Reviewer response for version 1

**DOI:** 10.21956/mep.19968.r30352

**Published:** 2020-12-28

**Authors:** Trevor Gibbs

**Affiliations:** 1 AMEE

This review has been migrated. The reviewer awarded 4 stars out of 5 With the increasing numbers of Letters to the Editor, seen in most journals now-days, this paper makes an interesting paper .I felt that it was well constructed and well written and gave an interesting approach to a very specific workshop.I think that this isa paper relevant to all those engaged in faculty development and mentoring programmes; I would be happy to recommend it

Reviewer Expertise:

NA

No decision status is available

# Reviewer report: Reviewer response for version 1

**DOI:** 10.21956/mep.19968.r30353

**Published:** 2020-04-07

**Authors:** Ken Masters

**Affiliations:** 1 Sultan Qaboos University

This review has been migrated. The reviewer awarded 4 stars out of 5 An interesting report on a workshop to teach physicians and students the intricacies of writing a letter to the editor.I am sure that many people do not know that there is a specific art to writing a letter to the editor, and the authors have done well to take their experience from previous workshops and build it successfully into this one.The success of the workshop is measured by both the standard evaluation and the publication of four letters in a reputable journal. (The editors of the journal must have been somewhat surprised to have received four letters relating to the same article :-)Given that journals are struggling for space, I would recommend that, in future, you have more articles, perhaps a maximum of two groups per article. Otherwise, you run the risk of having letters’ not being published, and then you would not know if it was because of the quality of the letter, of if the editors had decided that two letters on a particular article were enough.Overall, a good and solid description of a useful workshop.

Reviewer Expertise:

NA

No decision status is available

# Reviewer report: Reviewer response for version 1

**DOI:** 10.21956/mep.19968.r30351

**Published:** 2020-01-08

**Authors:** Felix Silwimba

**Affiliations:** 1 University of Lusaka

This review has been migrated. The reviewer awarded 5 stars out of 5 this is a good approach to sensitize and encourage practicing physicians to be writing and contributing to knowledge.

Reviewer Expertise:

NA

No decision status is available
